# Upper limb traumatic injuries: A concise overview of reconstructive options

**DOI:** 10.1016/j.amsu.2021.102418

**Published:** 2021-05-27

**Authors:** Marta Starnoni, Elisa Benanti, Andrea Leti Acciaro, Giorgio De Santis

**Affiliations:** a Department of Medical and Surgical Sciences, Division of Plastic Surgery, University of Modena and Reggio Emilia, Policlinico of Modena, Largo Pozzo 71, 41124, Modena, Italy; b Clinical and Experimental Medicine PhD Program, University of Modena and Reggio Emilia, Modena, Italy; c Department of Orthopaedics and Traumatology, Division of Hand Surgery and Microsurgery, University of Modena and Reggio Emilia, Policlinico of Modena, Largo Pozzo 71, 41124, Modena, Italy

**Keywords:** Upper limb reconstruction, Free flap, Pedicle flaps, Dermal substitutes, Upper limb traumas

## Abstract

Different options for upper limb reconstruction are described in literature: advancement or rotation flaps, regional flaps and free flaps are the most common. Local and regional flaps can represent the reconstructive options for small defects while large wounds require the use of free flaps or distant pedicled flaps. In case of large wound, the use of free flaps rather than distant pedicle flaps is usually preferred. To choose the best reconstructive option, it is essential for the surgeon to have a general overview about the different methods.

In this review the Authors will refer to the most commonly used methods to cover soft tissues injuries affecting the dorsum and the palm of the hand and the forearm (excluding fingers). The aim is to show all flap reconstructive options so as to support the inexperienced surgeon during the management of traumatic injuries of the upper limb.

## Highlights

• Reconstruction of traumatic injuries of the upper limbs can be challenging.
• Small defects can be covered by local and regional flaps while large wounds need the use of free or distant pedicled flaps.
• The literature shows different opinions whether to use pedicled flaps (regional or distant) or free flaps.
• Dermal substitutes can be considered in patients who are not suitable for flaps reconstruction.
• Patient related factors and surgical background can significantly interfere with the surgical reconstructive solution.

## 1 Introduction

The management of upper limb traumas may be challenging due to the involvement of several structures such as skin, bone, tendons, nerves, arteries and veins. A functional impairment of the limb can be reported. The most common causes of complex wound are road traffic and work-related accidents, as well as domestic injuries, burns, firearm accidents, etc. [1,2].

The hand and plastic surgeon have to provide a functional coverage of the wound with a good joint excursion. The coverage needs to be stable and long lasting allowing patient return to work as well as aesthetically pleasant [3]. The most challenging wounds may require amputation when is not possible to obtain a functional restoration because of wide and severe tissue damage. The age of the patient such as the characteristics of trauma, wound and surrounding tissue influence the reconstructive technique. Advancement or rotation flaps, regional flaps and free flap are the most common reconstructive options [4].

Defects can be classified according to anatomical location. In 2015, Rehim et al. modified the functional cutaneous units’ concept of Tubiana and introduced the functional aesthetic units and subunits of the hand that consider the principles of visual perception and anatomical aspects [5]. Ono et al. modified this concept by classifying dorsal and palmar soft tissues defects based on their characteristics: small (defect of a single surface of a metacarpal bone), medium (defect of two surfaces of a metacarpal bone or two adjacent surfaces of two metacarpal bones) and large (more than two metacarpal bone surfaces or non-contiguous defects) [6].

In this review we will refer to the most commonly used methods to cover defects on dorsum, on palm of the hand and on forearm.

## 2 Reconstruction by area

The Authors reviewed the available literature on wound coverage of dorsum and palm of the hand and of the forearm analyzing all the reconstructive options for each area. The aim is to provide a general overview of the coverage of hand wound in order to support the inexperienced surgeon in the management of damaged area reconstruction.

### 2.1 Dorsum of the hand

**Fig. 1:**
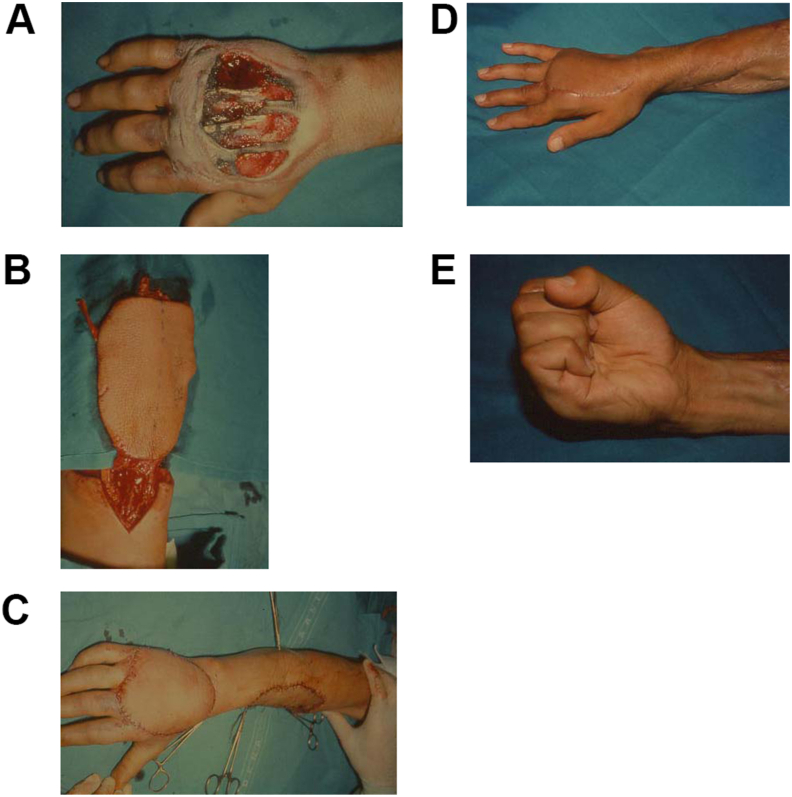
Reverse radial fascio-cutaneous forearm flap. Fig. 1aTraumatic injury of the dorsum of the hand. Fig. 1bFlap harvest. Fig. 1cFlap inset and coverage of the donor site with skin graft. Fig. 1dPost-operative result. e Tendons and joint function restoration.

In soft tissue coverage of the dorsum, tendons sliding must be preserved [7,8]. Several options are described including reverse radial forearm adipofascial, fasciocutaneous or fascial flap (Fig. 1), the posterior interosseous artery flap (PIA), groin flap or other abdominal pedicled flaps, dermal substitutes/skin graft or free flaps. For small dorsal defects, direct closure or local flaps are optimal. For medium-sized defects, the radial artery perforating flap (RAP), the ulnar artery perforator (UAP) or the PIA are usually used, whereas for large defects free flaps such as dorsalis pedis, anterolateral thigh flap (ALT) or abdominal distant flaps are preferred [6]. In the absence of a vascular injury to the same extremity, the most used method is the reverse pedicle radial forearm flap that can be also raised as a free flap thanks to its thin and pliable conformation which guarantees an adequate tendon sliding [9]. However, especially in middle-aged women with significant thickness of subcutaneous fat, it is better to harvest it as a pure fascial flap covered then by a skin graft [10]. Concerning the distant pedicled flaps, dorsal defects of the hand are covered with inferiorly pedicled base flaps: the superficial circumflex iliac artery flap (SCIA) and the superficial inferior epigastric artery flap (SIEA) [11]. The use of dermal substitutes followed by skin grafting is another option to consider for defects of the dorsum in selected cases.

### 2.2 Palm of the hand

A coverage with sturdy tissues that guarantee a good grip and support is essential for reconstruction of the palm. For small palmar defects it is possible to use conventional local flaps or conservative treatments such as artificial dermis. When deep vital structures are not exposed, palm has a high potential for second intention healing, whereas for medium-sized defects, forearm flaps such as the pedicled perforator flaps should be considered [6]. The groin flap is an excellent option if web space or finger stumps need to be covered. It brings copious tissues which can be useful in case of further procedures such as osteoplastic reconstruction of the thumb and toe transfer [12]. The serratus anterior flap can be used to cover the dorsum of the hand as well as the palm or the first webspace. Of note, the medialis pedis flap is able to restore the weightbearing of the palm [6].

### 2.3 Forearm

**Fig. 2:**
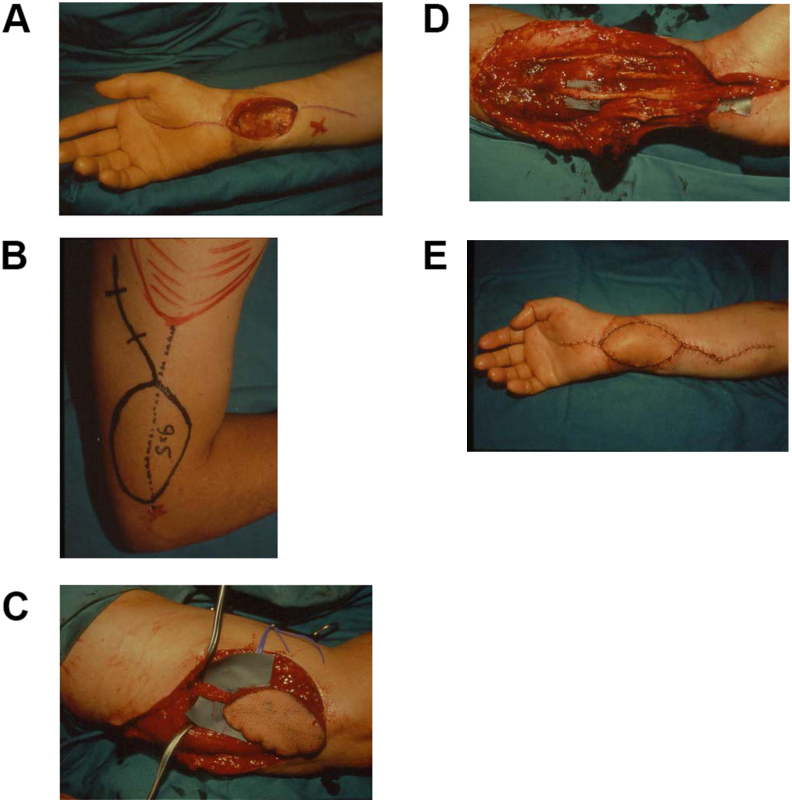
Lateral arm free flap. Fig. 2a. Defect of the volar region of the forearm. Fig. 2b. Preoperative flap mark. Fig. 2c. Flap harvest. Fig. 2d. Microvascular anastomosis. Fig. 2e. Post-operative result.

Free flaps or pedicled distant flaps are generally required to cover large wounds of the forearm. The ALT is a workhorse in the reconstruction of the upper limb thanks to its long pedicle, simple dissection, possibility of thinning up to 3 mm, and minimum morbidity of the donor site. A sensate flap as well as a composite flap with muscle or fascia can be harvested. Two equips, both in the donor and recipient site, can simultaneously work with the patient in a supine position [13]. The lateral arm flap (Fig. 2), supplied by the posterior collateral radial artery, has to be mentioned for forearm reconstruction. It can be used both as pedicled or free flap and it can be raised as a sensate flap with the posterior brachial cutaneous nerve reinnervation [14]. Among the distant pedicled flaps the paraumbilical perforator (PUP) is the best choice for forearm wounds, due to patient comfort during the post-operative time before division [15].

## 3 Discussion

Small defects are covered by local and regional flaps while large wounds need the use of free flaps or distant pedicled flaps [16]. Limits of local flaps for coverage of large defects are represented by the poor expandability of the donor site, the reduced flap's range of motion and the frequent damage of the surrounding tissue with possible compromising of transfer vitality [13]. In case of large wounds, the use of free flaps rather than distant pedicle flaps is the choice [16]. In order to choose the best reconstructive option, several algorithms have been proposed [16]. Chim et al. proposes an algorithm based on the specific characteristics of patient and wound, taking into consideration the preparation of wound bed, the area of injury and the replacement like to like [16].

Free flaps provide the best coverage in cases of severe injury of upper limb. The microvascular flaps bring good skin coverage and can be combined with fascia, muscle, bone, and tendons, providing healthy tissues and facilitating vascular growth from the surrounding tissues [17]. The blood supply has the advantage of improving bone healing and resistance to infections [13]. Moreover, these flaps have the advantage of requiring few days of hospitalization and patients don't have the discomfort of the attached limb to the abdomen. Complete reconstruction in a single stage allows early mobilization, reduces fibrosis and avoids stiffness [18]. Of note, mobilization should begin as soon as possible to prevent joint stiffness, tendon adhesion and soft tissue contracture which can compromise long-term outcomes [19]. Fasciocutaneous flaps have a better color match and a greater choice of the donor area compared to muscular flaps. In selected cases, perforator flaps can be an alternative good option because of the lower morbidity of the donor site and the less bulky aspect [16].

In the 1970s and 80s the distant pedicled flaps such as groin and abdominal flaps were the workhorses for hand and forearm reconstruction. These flaps have well-known disadvantages such as the need of flap division, the patient discomfort and emotional stress, an increased hospitalization with higher costs, the need of a debulking, as well as joints stiffness and long physiotherapy sessions. Furthermore, their use is limited by the rotation arc, the extension and the position of the injury [11,13]. Nevertheless, when well executed distant pedicled flaps can be even better than free flaps in long-term outcomes [20], especially in patients with comorbidities. They are fast and easy to raise and in obese patients they are certainly thinner than other flaps [21]. Abdominal flaps are versatile and do not require technical skills or microsurgical instruments. They can be cost effective but proper technical refinements should be performed. A narrow base allows a good inset without bunching and unevenness of the flap. In order to have a more comfortable position it is mandatory to keep an adequate length of the pedicle allowing mobility. A dedicate and prompt post-operative rehabilitations therapy is fundamental. The vascularization of the injured limb is not potentially compromised because there is not vessels manipulation in comparison with free flap. Moreover, they can be thinned during the second surgical step almost up to the subdermal level [21,22]. The training period, the attention to details and the ability to perform are certainly lower than the microsurgical ones and give the surgeon a greater level of confidence when facing with a complex defect [20]. Pedicled flaps should always be considered because they can allow further reconstructive options. In case of large injuries with bone loss and a single vessel limb, the pedicled flap can be used to cover the soft tissue defect allowing a future bone reconstruction with a free flap [20]. In many parts of the world, distant pedicled flaps are still workhorse in the management of upper limb reconstruction and are unlikely to be displaced by free flaps [23]. Free flaps are used only when other options are not available and when the defect cannot be covered otherwise. Free flaps provide coverage in a single stage; however, they require experience, a long operating time, facilities, a learning curve and they have a potential for failure [4,24,25]. Distant pedicled flaps can be considered a good coverage option and preferred to free flaps in specific situations of large wounds that cannot be covered by loco-regional flaps. Common indications are represented by poor receiving vessels, extended scars, and severe comorbidities. The demanding of a toe transfer as a second surgical time may require a pedicle flap for prevention of contractures, preservation of a stump and recipient vessels for a second microsurgical step [26]. In case of electrical burns injuries, recipient vessels are often damaged. The use of free flaps can lead to hand ischemia if thrombosis occurs [11]. However, some authors prefer the use of free flaps because a burned hand is prone to stiffness and free flaps allow an early mobilization [27]. The coverage of metacarpal heads is another described indication [28]. Nevertheless, all these indications can be disproved and reviewed in favor of microsurgery, although free flaps can have a greater number of postoperative complications in the hand of unskilled surgeon. Certainly, pedicled abdominal flaps find their main indications in cases in which the patient's health is critical (as in polytrauma), the vessels are damaged, if other surgical procedures are planned such as toe transfer or fibula flap or when the general anesthesia and long surgeries are contraindicated (pregnancy), as well as in case of microsurgical flap failure and in the setting of limited economic and technical resources [20,21].

A possible approach to upper limb wounds could be the initial application of dermal substitutes or vacuum therapy and wait for granulation to occur. This can be performed in all situations in which the defect is not so small to be covered with local flaps and not so large to need a free flap or a distant pedicled flap. Bioengineering products are a valid option to consider in patients who are not suitable for flaps reconstruction. In 1981 Burke et al. described artificial dermal substitutes composed by a layer of silicone epidermis and a dermis of porous collagen chondroitin 6-sulfate fibrillar, used for extensive burns treatment [29]. Dermal substitutes are a heterogeneous group of wound coverage materials that help in closing wounds and replace skin functions, sometimes temporarily, sometimes permanently depending on their characteristics [30]. These substitutes provide many biological and physiological properties of human dermis, can promote tissue growth and optimize healing conditions [31]. Dermal substitutes can be used in the hand to cover critical structures such as tendons without paratenon, cartilage without perichondrium and bone without periosteum. The complete bio-integration requires a well vascularized wound bed free from infections [32]. Therefore, the use of dermal substitutes should be considered as an additional option, especially if local tissues are damaged or unavailable. In case of soft tissue damage with exposed tendon and absent paratenon, skin substitute can be considered a convenient and efficient option for immediate tendon coverage in terms of tendon function restoration and good cosmetic results [33]. For small wounds the use of free flap is not convenient as it brings additional costs and resources, and surrounding tissues are often unavailable: in these cases, dermal substitutes can be a valid alternative [34]. The simplicity of the procedure and a minor donor site morbidity are the most important advantages. Moreover, favorable cosmetic and functional outcomes have been reported with the use of dermal substitutes for deep defects of the hand after burns, tumors excision and injuries of fingers that cannot be covered with local flaps [35]. Certainly, the high initial cost of dermal substitutes could be a disincentive to use them limiting their availability. This is true in developing countries, while more economically advanced countries are likely to buy dermal substitutes. In our knowledge, there is only one study on the cost analysis that compares the total costs derived from the use of dermal substitutes vs. total skin graft costs for small burns treatment: the costs of dermal substitutes were higher, but not statistically significant; indirect costs such as the duration of hospitalization and overheads have been the most important factors in influencing the total cost of treatment [32]. The potential use of dermal substitutes in difficult wounds with deep structures exposure leaves an open chapter that could avoid more complex procedures and cause less morbidity to patients [36].

Of note, the surgical background can significantly interfere with the surgeon's choices in traumatic emergencies as well as in elective procedures both in hand and plastic surgery [37]. Different surgical aspects have to be taken into considerations when facing with the patient:

- detailed knowledge of the topic and all surgical solutions [38];
- technical possibilities according to hospital class (hub or spoke center) and epidemiologic challenges such as emerging COVID-19 pandemic [[39], [40], [41], [42]];
- required instruments to perform microsurgery [[43], [44], [45], [46], [47]];
- use of new technologies, innovative surgical methods and unconventional devices [[48], [49], [50], [51], [52]];
- possibility to refer to skilled consultants;
- possibility to work in multi-equip with different specialists [[53], [54], [55], [56], [57], [58]];
- selection of high-risk surgical wound complications patients throughout available scores [59,60];
- the help of skilled health professionals able to early detect possible complications and promptly start proper care and close follow-up [61,62].

These aspects are crucial to define the context of the patient treatment. Technical surroundings are extremely different from one care center to another. The healthcare background is fundamental when choosing among different surgical options. The best surgical solution available in a hospital could be the worst if performed in another health center [2,63,64]. Despite several studies and innovative techniques, we have not yet reached a scientific conclusion on the best type of coverage [65]. Beyond the possible indications of different centers, variables that play an important role in the decision are patient related and surgeon dependent as well as depending on the economic possibilities and facilities of the different hospitals [66]. The patient's age, employment, other injuries and future plans are factors to consider: in the planning of reconstruction it is good to have in mind from the beginning all the possible surgical steps [67]. Concerning the patient age there are conflicting opinions between those who prefer distant flaps because of the patients comorbidities and those who prefer free flaps for the lower risk of joints stiffness: the groin flap is generally contraindicated in elderly patients because it could predispose to shoulder stiffness, and in young children because of the difficult cooperation [10]. A decision-making algorithm for selecting an ideal flap for a particular hand defect requires experimental considerations on functional outcome, aesthetic appearance, donor site morbidity and patient satisfaction. To select the best and most appropriate flap, more studies are needed with scientific evidence that can compare the different outcomes [6].

## 4 Conclusion

In order to choose the best reconstructive option of the upper limb, several algorithms have been proposed, but it seems that surgeon experience can represent a useful help. Thoughts gleaned from the wide experience of a surgeon represent important evidence-based advice that can be essential for the decision-making process.

## Author contribution

Marta Starnoni: study concept, data interpretation, writing the paper. Elisa Benanti: study concept, data interpretation, writing the paper. Andrea Leti Acciaro: data collection. Giorgio De Santis: study concept, data interpretation, writing the paper.

## Guarantor

Marta Starnoni, Elisa Benanti; Andrea Leti Acciaro; Giorgio De Santis.

## Trial registry number

Nothing to declare.

## Ethical approval

Nothing to declare.

## Sources of funding

Nothing to declare.

## Provenance and peer review

Not commissioned, externally peer reviewed.

## Declaration of competing interest

The authors declares that there is no conflict of interest regarding the publication of this paper.
